# When aggregation-induced emission meets protein aggregates

**DOI:** 10.1093/nsr/nwab013

**Published:** 2021-01-25

**Authors:** Sicheng Tang, Songtao Ye, Xin Zhang

**Affiliations:** Department of Chemistry; Department of Chemistry; Department of Chemistry; Department of Biochemistry and Molecular Biology, The Pennsylvania State University, USA

## Abstract

There is an unmet demand for research tools to monitor the multistep protein aggregation process in live cells, a process that has been associated with a growing number of human diseases. Recently, AIEgens have been developed to directly monitor the entire protein aggregation process in test tubes and live cells. Future application of AIEgens is expected to shed light on both diagnosis and treatment of disease rooted in protein aggregation.

Protein misfolding and aggregation have been associated with a number of human diseases. As a multistep process (Fig. 1a), protein unfolding yields misfolded intermediates as either monomers or oligomers, which evolve into insoluble aggregates as amorphous aggregates or amyloid-β fibrils. Detection of protein aggregates is of great interest as it could generate unprecedented insights into biogenesis, regulation and pathology of protein aggregation. Previous efforts using fluorescence detection have generated various fluorophores with different fluorescence turn-on mechanisms and a wide spectrum coverage. However, the π-rich conjugated system of these traditional fluorophores often suffers from aggregation caused quenching (ACQ). Based on the tetraphenylethene (TPE) scaffold, aggregation induced emission (AIE) fluorophores were introduced by Tang and co-workers to avoid the ACQ feature as AIE fluorophores (AIEgens) exhibit strong fluorescence when aggregated because of restricted intramolecular motion (RIM) [1]. In recent years, AIEgens have been developed to detect protein unfolding, oligomerization and aggregation. These results also potentiate AIEgens as a powerful means to modulate this important biological process for the benefit of human health.

**Figure 1.**
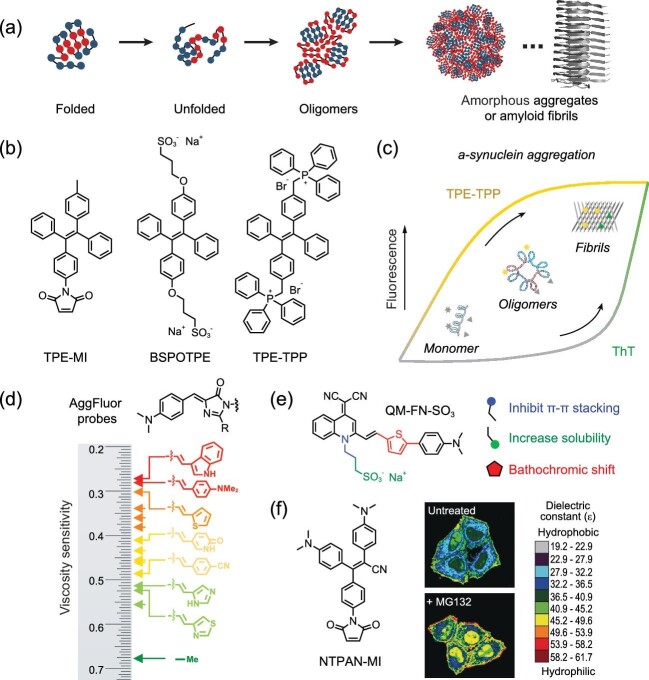
Detecting protein aggregation using AIEgens. (a) The multistep process of protein aggregation. (b) Examples of TPE-based AIEgens to detect protein aggregation. TPE-MI: tetraphenylethene maleimide. BSPOTPE: 1,2-bis[4-(3-sulfonatopropoxyl)phenyl]-1,2-diphenylethene salt. TPE-TPP: bis(triphenylphosphonium) tetraphenylethene. (c) a-synuclein aggregation monitored by TPE-TPP. (d) Structure of AggFluor probes. (e) Converting ACQ probes to AIEgens. (f) Ratiometric imaging of protein aggregation using NTPAN-MI. (d) was modified from Wolstenholme *et al.* [8]; (f) was modified from Owyong *et al.* [10].

It is generally recognized that protein aggregation is initiated with a partial unfolding of the folded proteins. Therefore, studies on dynamics of the unfolding process are vital for the early detection of protein aggregation. To this end, unfolding-induced fluorescence quenching is achieved by a water soluble TPE-based AIEgen to visualize the unfolding events of human serum albumins (HSAs) in the presence of guanidine hydrochloride (GndHCl) [2]. The fluorescence is activated in folded HSA when such a fluorophore is docked onto the interdomain hydrophobic region. The unfolding process, however, frees these water-soluble AIE fluorophores into the aqueous solvent and quenches fluorescence in a stepwise manner. The work from Hong *et al.* settled the controversial studies of the unfolding process of HSA by revealing a three-step conformational change as formation of unfolded coils, molten globule intermediates and the final domain separation. Conversely, unfolding-induced fluorescence activation is also used to report on protein unfolding and proteostasis stress using TPE maleimide (TPE-MI, Fig. 1b) [3]. TPE-MI remains non-emissive either in solution or aggregate state and can be activated by two criteria: (1) the removal of the quenching effect by conjugating MI to thiol and (2) restricted intramolecular rotation (RIR) of TPE. When intracellular proteins unfold, exposed free cystines and hydrophobic cores collectively activate the fluorescence of TPE-MI. Because of the unique behavior of TPE-MI, it bypasses the false positive signal from accessible thiols on folded protein surface and non-protein thiols such as glutathione, offering a unique opportunity to report on protein unfolding in live cells.

Recent studies have revealed that toxic misfolded protein oligomers could undermine the cellular proteostasis network and further evolve into protein aggregates, in particular, amyloid fibrils. In this regard, a biocompatible AIEgen, 1,2-bis[4-(3-sulfonatopropoxyl)phenyl]-1,2-diphenylethene salt (BSPOTPE), is developed to report insulin amyloidogenesis [4]. BSPOTPE exhibits significant fluorescence activation that correlates with insulin nucleation, elongation and equilibrium phases, thereby enabling the detection of misfolded oligomers and the evaluation of amyloidogenesis kinetics. More interestingly, BSPOTPE is found to be a potent *in*-*situ* kinetic stabilizer that inhibits the insulin amyloid cascade with a dose-dependent manner. Furthermore, an AIE probe TPE-TPP is developed to distinguish the misfolded oligomers of α-synuclein, without affecting its fibrillation (Fig. 1b) [5]. Distinct from commonly used Thioflavin-T (ThT), which only detects mature fibrils, TPE-TPP exhibits a significant fluorescence enhancement and a shorter lag phase when monitoring *α*-synuclein fibrillation (Fig. 1c). This quick fluorescence activation indicates that TPE-TPP is able to detect the intermediate misfolded oligomers during the process of fibrillation. In addition to BSPOTPE and TPE-TPP, several aggregation-detecting AIEgens have been developed to extend the spectral coverage of the probes [6] as well as the variety of proteins [7].

Besides the most commonly used TPE scaffold, novel AIEgens have also been reported to detect protein aggregation. The chromophore of green fluorescent protein, 4-hydroxybenzylidene-imidazolinone (HBI), is modulated to detect and differentiate misfolded oligomers and protein aggregation by sensing the local viscosity changes [8]. These fluorophores, named AggFluor, comprise a series of AIEgens that exhibit a broad coverage of sensitivity towards local viscosity (Fig. 1d). Combined with the notable self-labeling protein tagging technologies, AggFluor enables a multi-color imaging capacity which is suitable to address complex cellular events including regulation of protein aggregation and membrane-less organelles. Strategies have also been proposed to convert existing ACQ probes to AIEgens through introduction of a bulky ethyl group to prevent π–π stacking [9]. Further functionalization results in extended wavelength to near infrared region for compound QM-FN-SO_3_ (Fig. 1e), thus enabling a high-fidelity fluorogenic detection of amyloid-β in living mice. Finally, a novel class of push-pull fluorophores (NTPAN-MI) that exhibit strong solvatochromism are reported by changing one phenyl ring of TPE-maleimide with an electron-withdrawing cyano group (Fig. 1f) [10]. These newly designed molecules have helped to reveal the intracellular unfolded protein load and map the intracellular polarity profile when cells are under stress (Fig. 1f). The outcome from this work suggests a more hydrophilic local environment in the nucleus under various stresses, which relates to multiple nucleus functions during the stress response.

As a powerful molecular toolbox to study protein aggregation, the broad chemical space of AIEgens potentiates exciting applications via the development of novel chemical scaffolds and structures. For instance, imaging of protein aggregation in living organisms or tissue samples can be achieved if the spectral coverage of AIEgens is systematically tuned to the range of near-infrared (NIR-I and NIR-II) to minimize background signal and maximize tissue penetration. Beyond imaging as a potential diagnostic application, the bright future of AIEgens lies in their capacity to serve as imaging-guided theranostic agents. Photodynamic therapy (PDT) based on photosensitizers under light irradiation can go through intersystem crossing to the triplet state, followed by the generation of ^1^O_2_ for efficient ablation of unhealthy cells. AIEgens outperform traditional photosensitizers by avoiding ACQ, which would significantly reduce ^1^O_2_ generation in the aggregate state. The efficiency of intersystem crossing can be further improved by reducing the energy gap between singlet and triplet states. Photothermal therapy (PTT) is another efficient way for AIEgens to ablate unhealthy cells affected by protein aggregation. The energy dissipation of AIEgens can be adjusted to the form of heat by adding bulky alkyl chains to decrease intermolecular interaction. The promoted non-radiative decay process would be able to conduct PTT with high photothermal conversion efficiency and strong photoacoustic signal. Given the rapid development in this field, AIEgens hold promise for potential treatments for diseases rooted in protein aggregation.

## References

[1] Hong Y , Lam JWY, Tang BZ. Chem Soc Rev 2011; 40: 5361–88. 10.1039/c1cs15113d 21799992

[2] Hong Y , Feng C, Yu Y et al. Anal Chem 2010; 82: 7035–43. 10.1021/ac1018028 20704392

[3] Chen MZ , Moily NS, Bridgford JL et al. Nat Commun 2017; 8: 474. 10.1038/s41467-017-00203-5 28883394 PMC5589734

[4] Hong Y , Meng L, Chen S et al. J Am Chem Soc 2012; 134: 1680–9. 10.1021/ja208720a 22191699

[5] Leung CWT , Guo F, Hong Y et al. Chem Comm 2015; 51: 1866–9. 10.1039/C4CC07911F 25526628

[6] Yang Y , Li S, Zhang Q et al. J Mater Chem B 2019; 7: 2434–41. 10.1039/C9TB00121B 32255120

[7] Ding S , Yao B, Schobben L et al. Molecules 2019; 25: 32. 10.3390/molecules25010032 PMC6982923 31861868

[8] Wolstenholme CH , Hu H, Ye S et al. J Am Chem Soc 2020; 142: 17515–23. 10.1021/jacs.0c07245 32915553 PMC8088285

[9] Fu W , Yan CX, Guo ZQ et al. J Am Chem Soc 2019; 141: 3171–7. 10.1021/jacs.8b12820 30632737

[10] Owyong TC , Subedi P, Deng J et al. Angew Chem Int Ed 2020; 59: 10129–35. 10.1002/anie.201914263 31826303

